# Supplementary material for: Immune Transcriptome Study of Human Nucleated Erythroid Cells from Different Tissues by Single-Cell RNA-Sequencing
Source: Cells. 2022 Nov 9;11(22):3537. doi: 10.3390/cells11223537 (PMC9688070; doi:10.3390/cells11223537)
Supplement: Supplementary file 1 [file cells-11-03537-s001.zip › file S2-Pseudotime trajectory inference of NECs from each tissue of origin.pdf]

Pseudotime trajectory inference of NECs from each tissue of origin

We examined NECs transcriptome by inferring their differentiation in pseudotime using Monocle. We observed that for adult bone marrow NECs pseudotime plot's (Figure S1a) components were mainly driven by *ALAS2, ARG1, HLA-A, CD36, LGALS3* and *SLC25A37* gene expression. We observed that *ARG1* gene expression and *LGALS3* gene expression were inversely correlated with *CD36* gene expression (Figure S1b).

a)

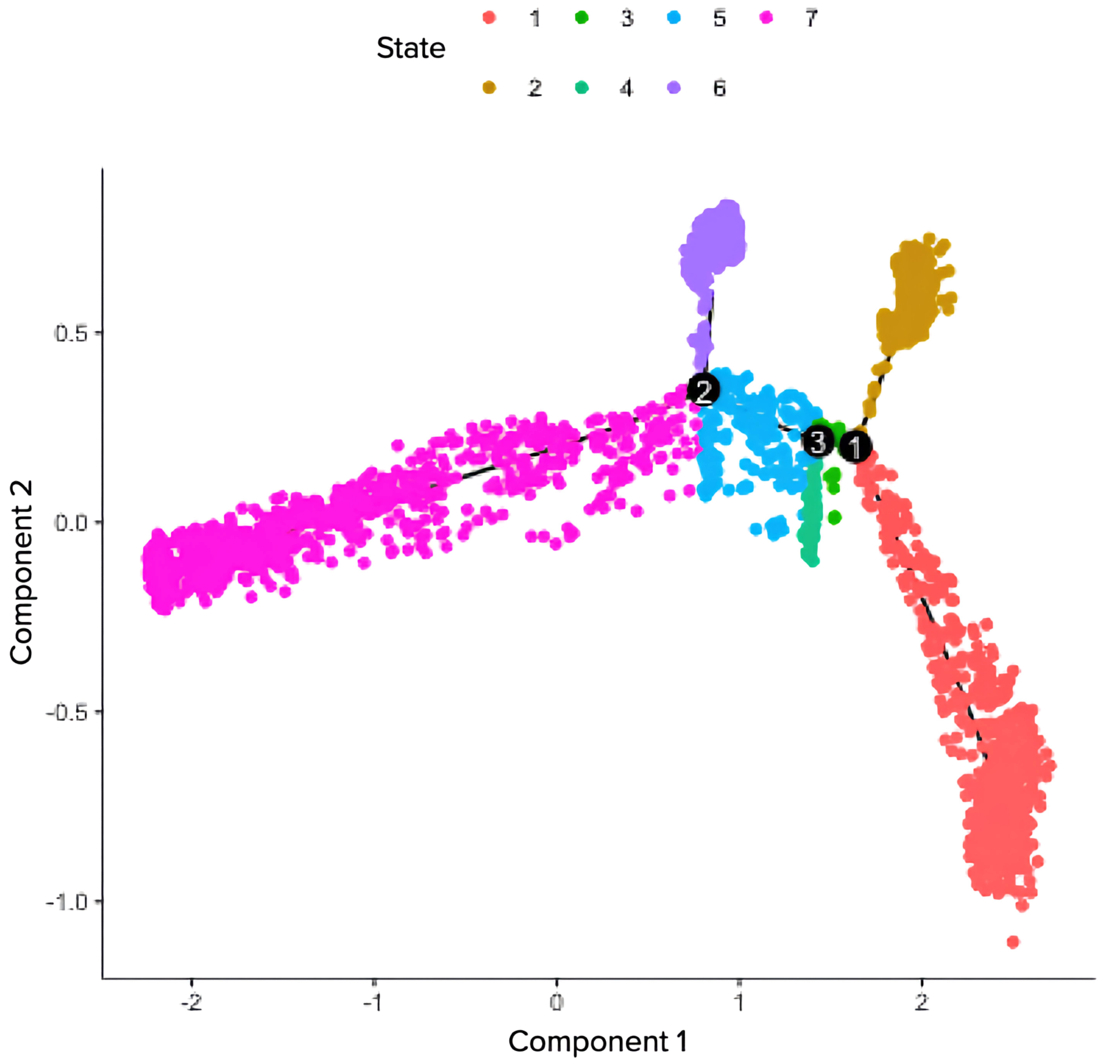

b)

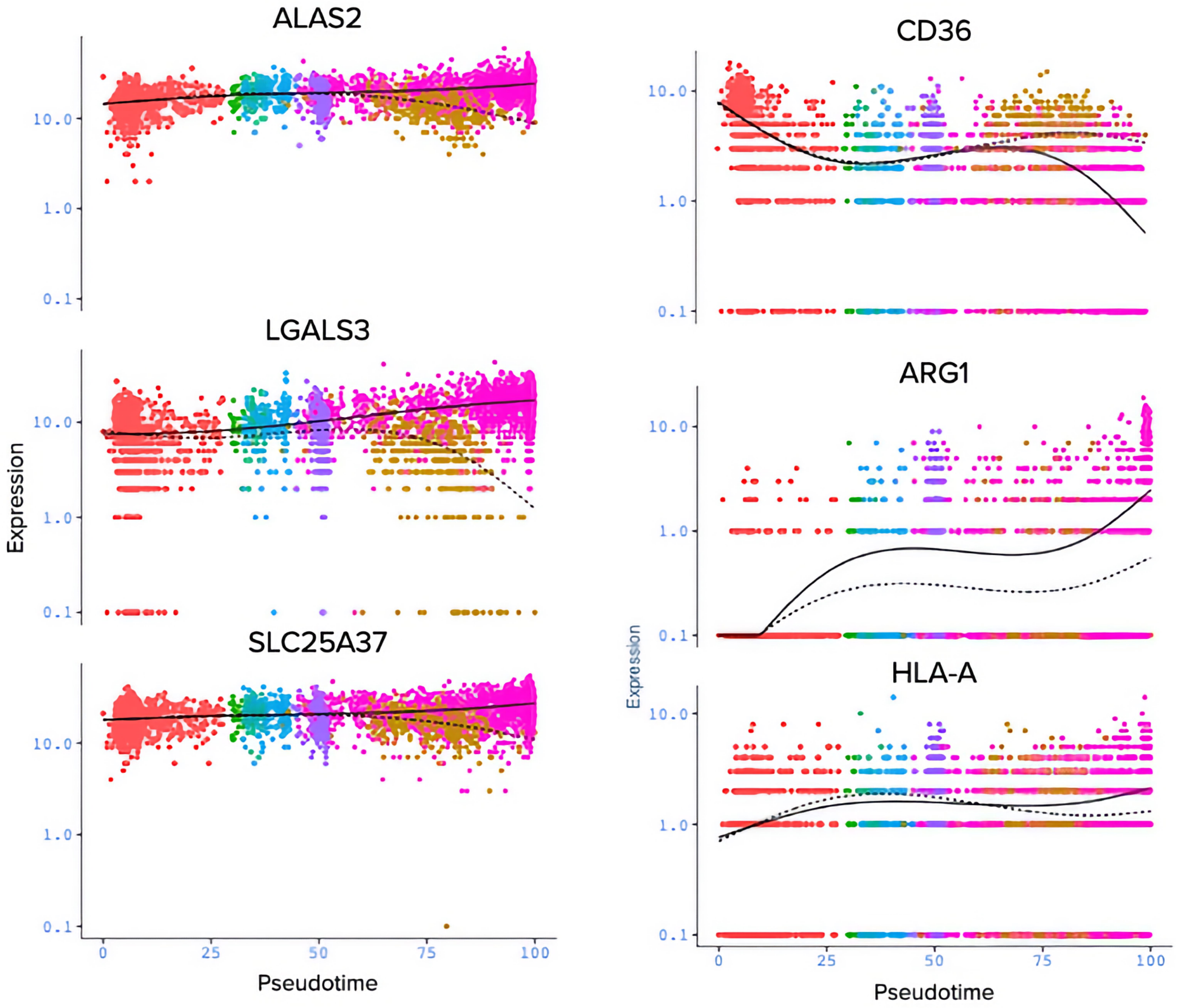

Figure S1. Differentiation estimation of adult bone marrow NECs in pseudotime: a) Monocle plot b) Kinetics of pseudotime-driving genes**.**

Cord blood NECs' pseudotime plot's (Figure S2a) components were mainly driven by *ALAS2*, *ARG1*, *HLA-A* and *CD36* gene expression. We observed that *ARG1* gene expression and CD36 gene expression were inversely correlated (Figure S2b).

a)

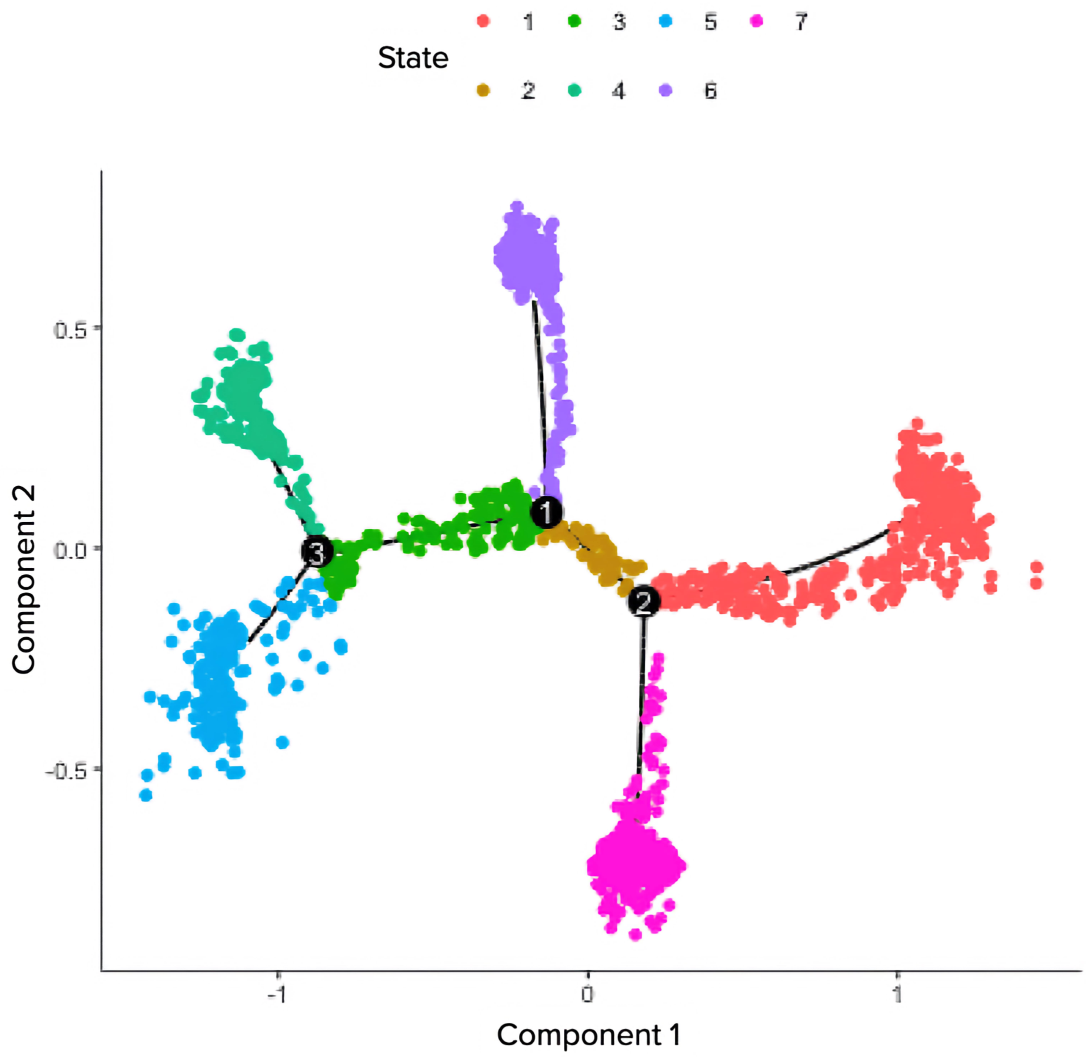
State
1
2
3
4
5
6
7
Component 2
0.5
0.0
-0.5
Component 1
-1
0
1

b)

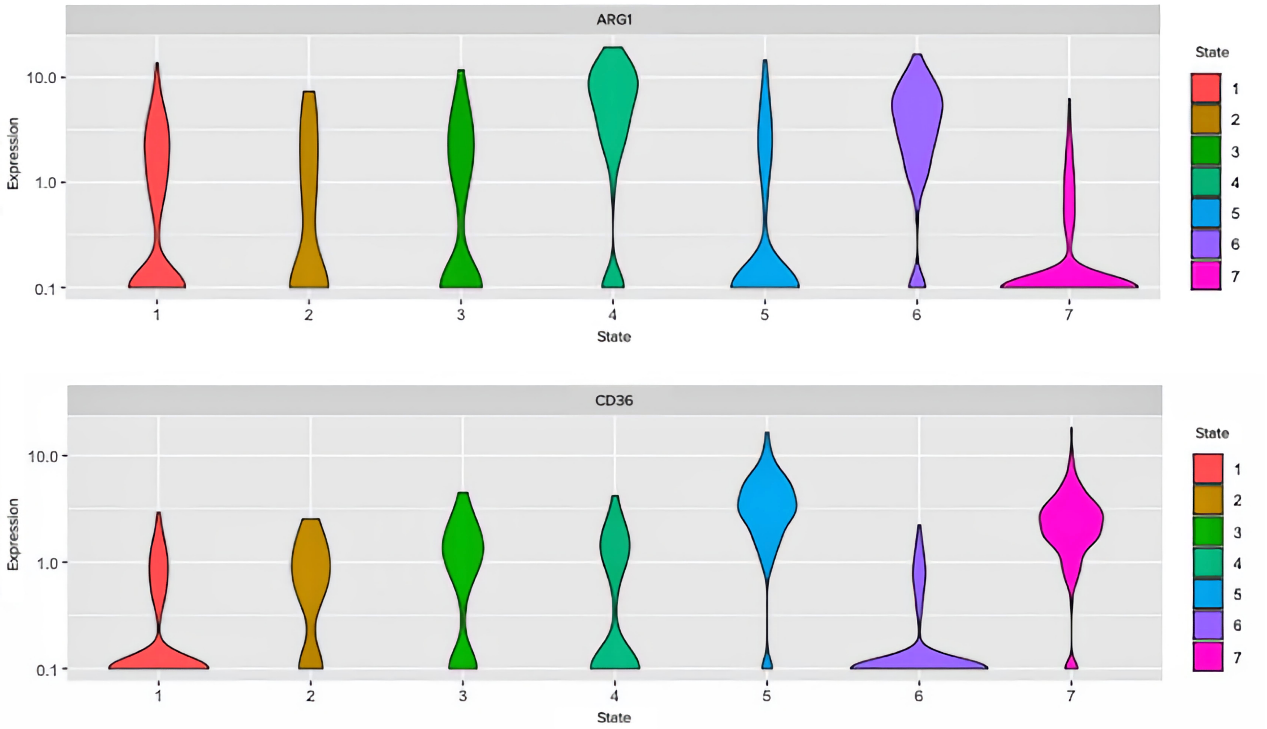
ARG1
Expression
10.0
1.0
0.1
State
1
2
3
4
5
6
7
CD36

Figure S2. Differentiation estimation of cord blood NECs in pseudotime: a) Monocle plot b) violin plots of the main pseudotime-driving genes *ARG1* and *CD36*.

Fetal liver parenchyma NECs' pseudotime plot's (Figure S3a) components were mainly driven by *ALAS2*, *HLA-A*, *CD36* and *LGALS3* gene expression. We observed that *LGALS3* gene expression and *CD36* gene expression were inversely correlated (Figure S3b).

a)

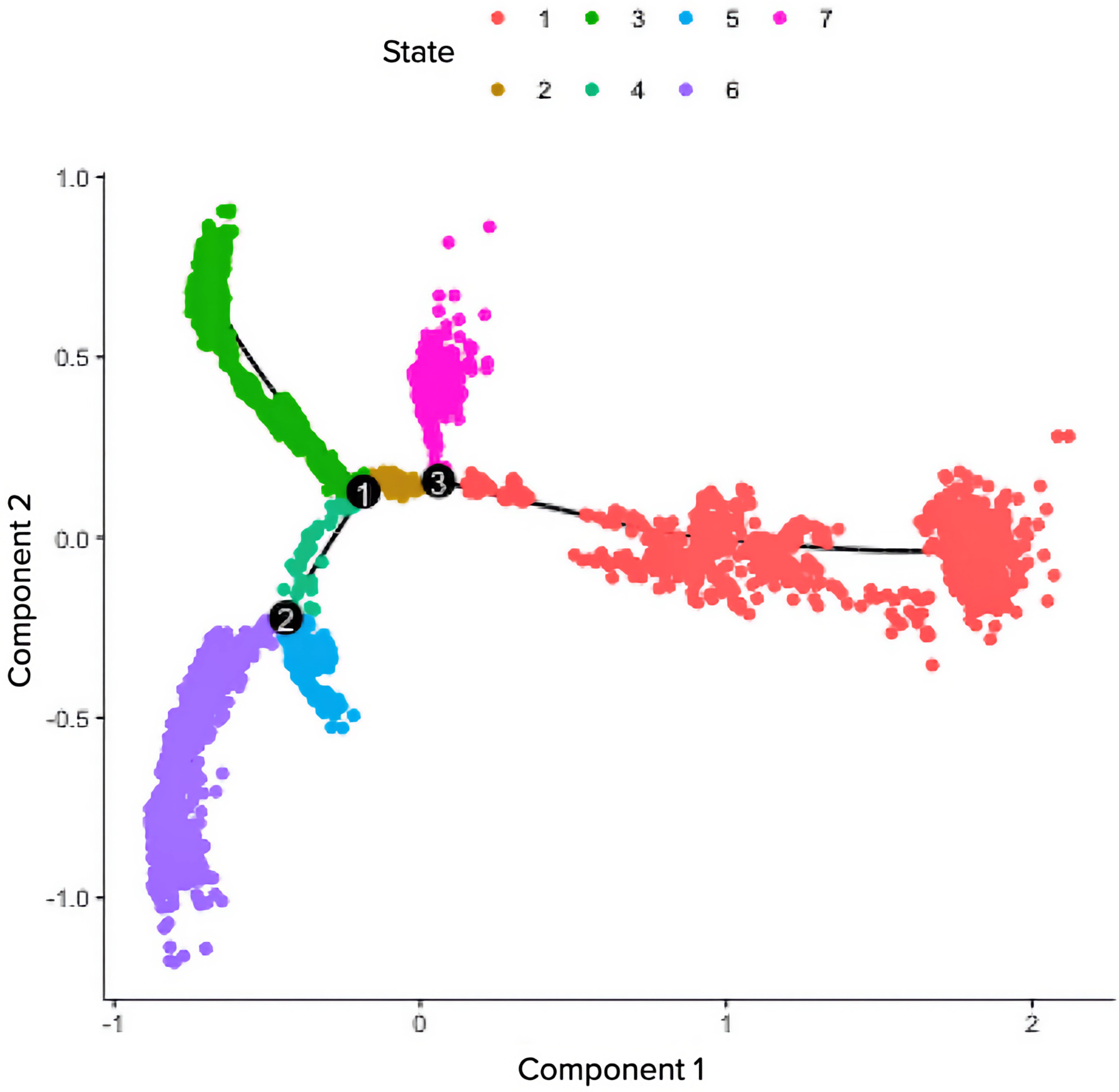

b)

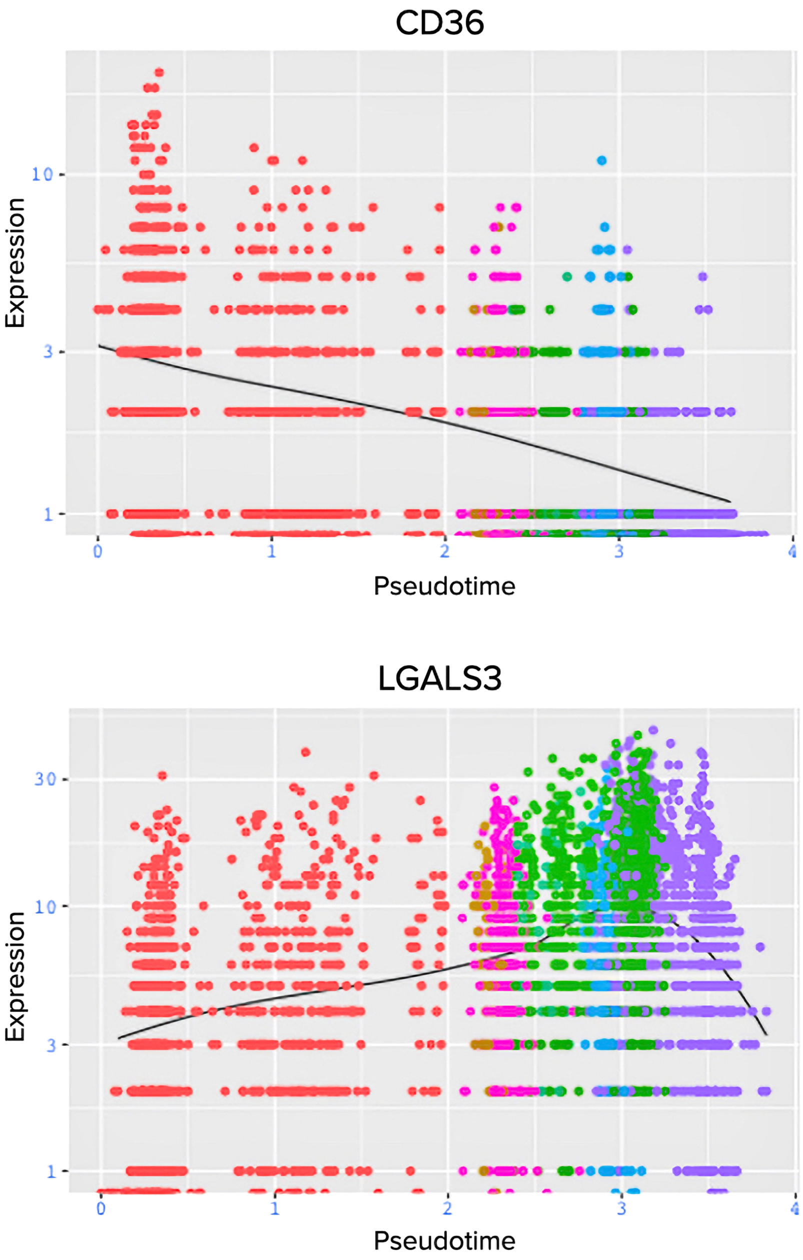

Figure S3. Differentiation estimation of fetal liver parenchyma NECs in pseudotime: a) Monocle plot b) Kinetics of pseudotime-driving genes *CD36* and *LGALS3*.
