# Supplementary figures and images for: Immune Transcriptome Study of Human Nucleated Erythroid Cells from Different Tissues by Single-Cell RNA-Sequencing
Source: Cells. 2022 Nov 9;11(22):3537. doi: 10.3390/cells11223537 (PMC9688070; doi:10.3390/cells11223537)

UMAP 2

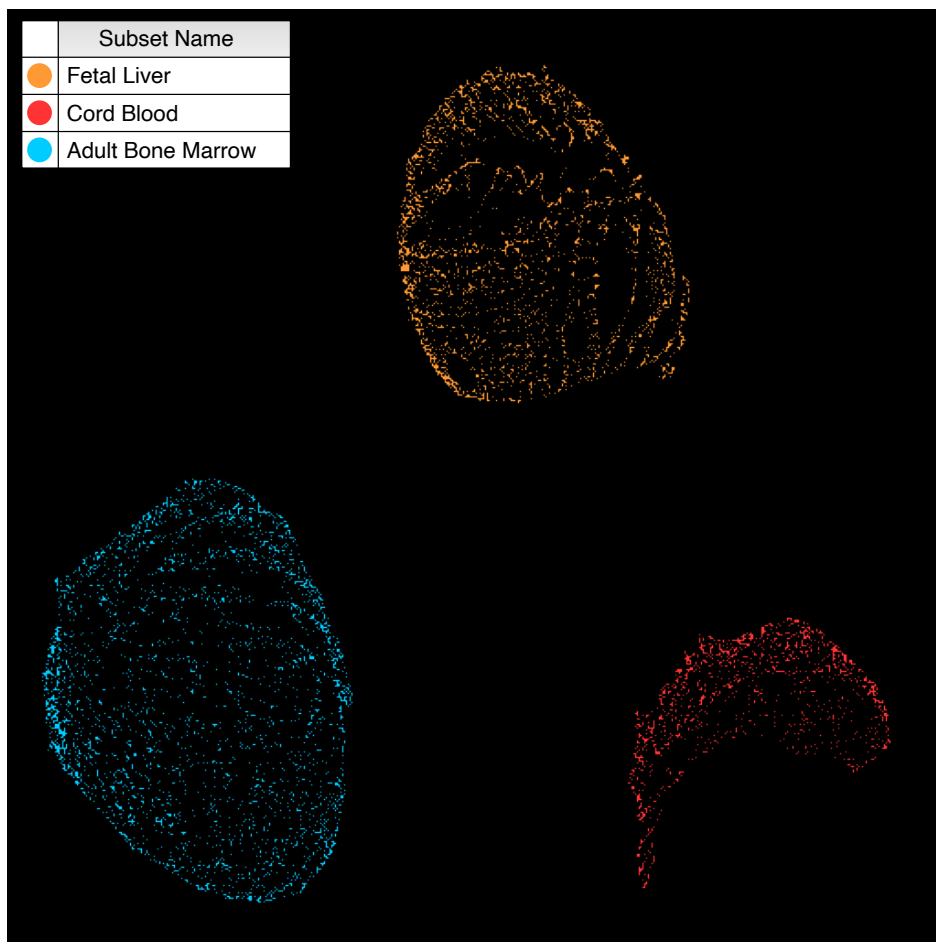

UMAP 1

IGHE secreted

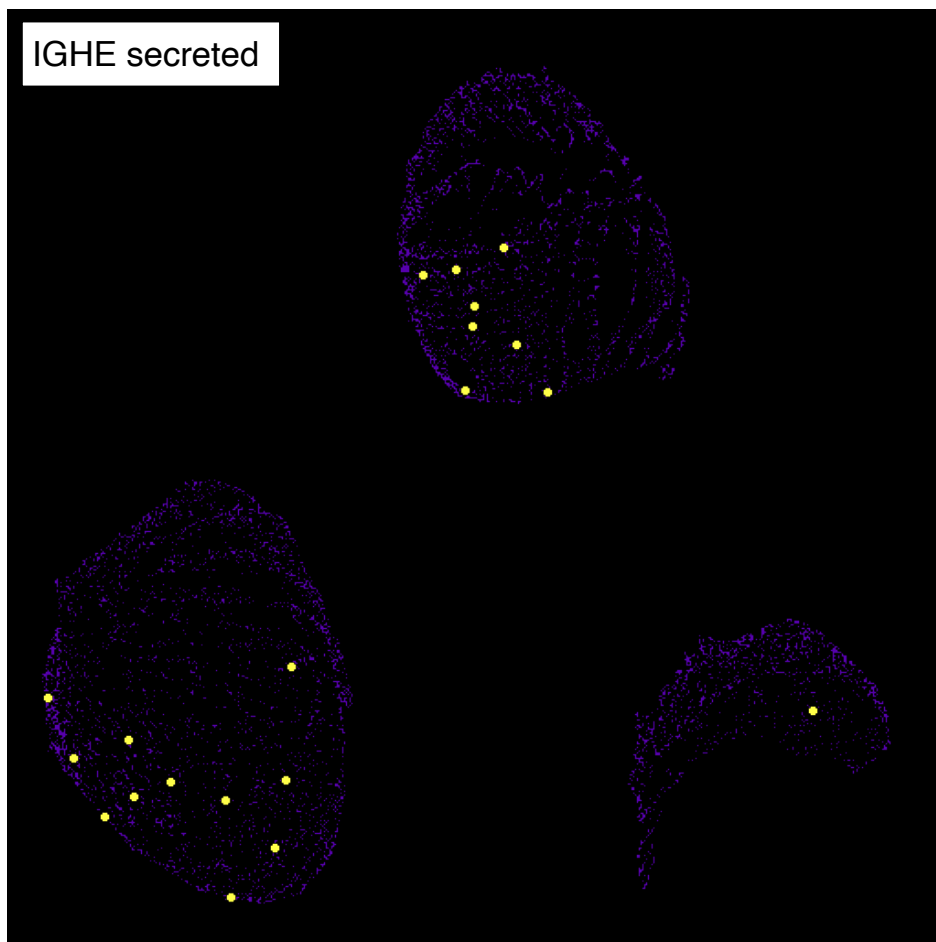

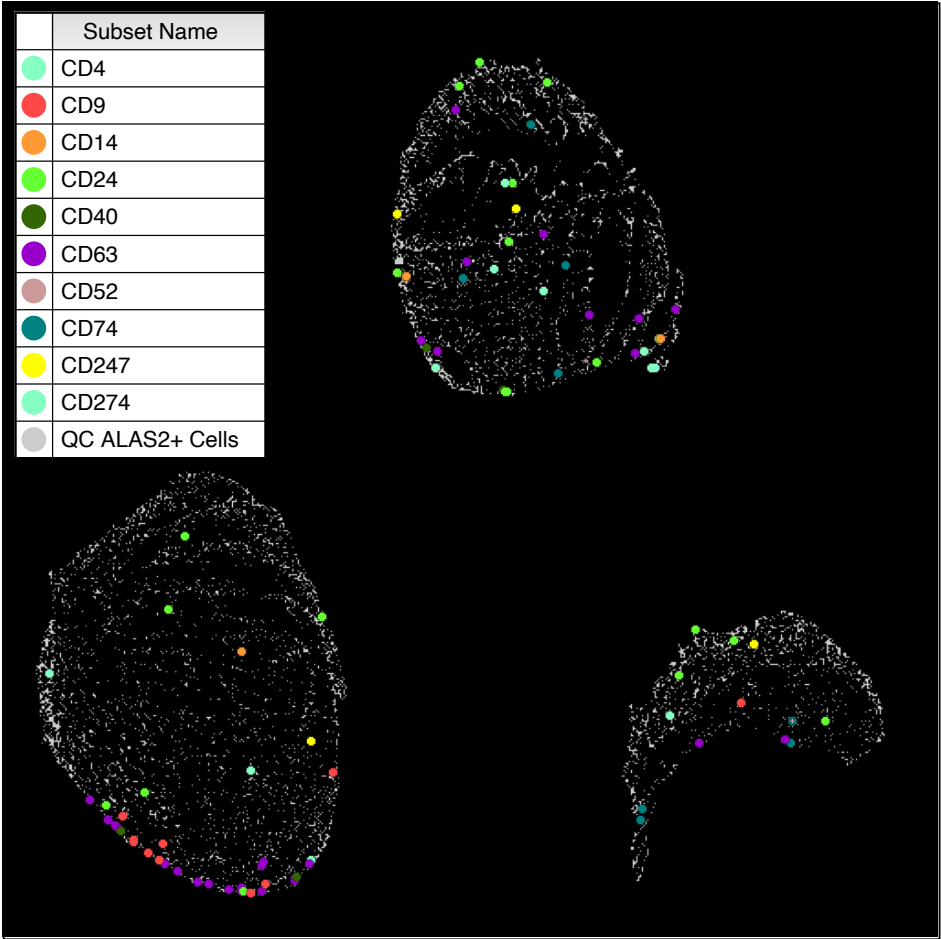

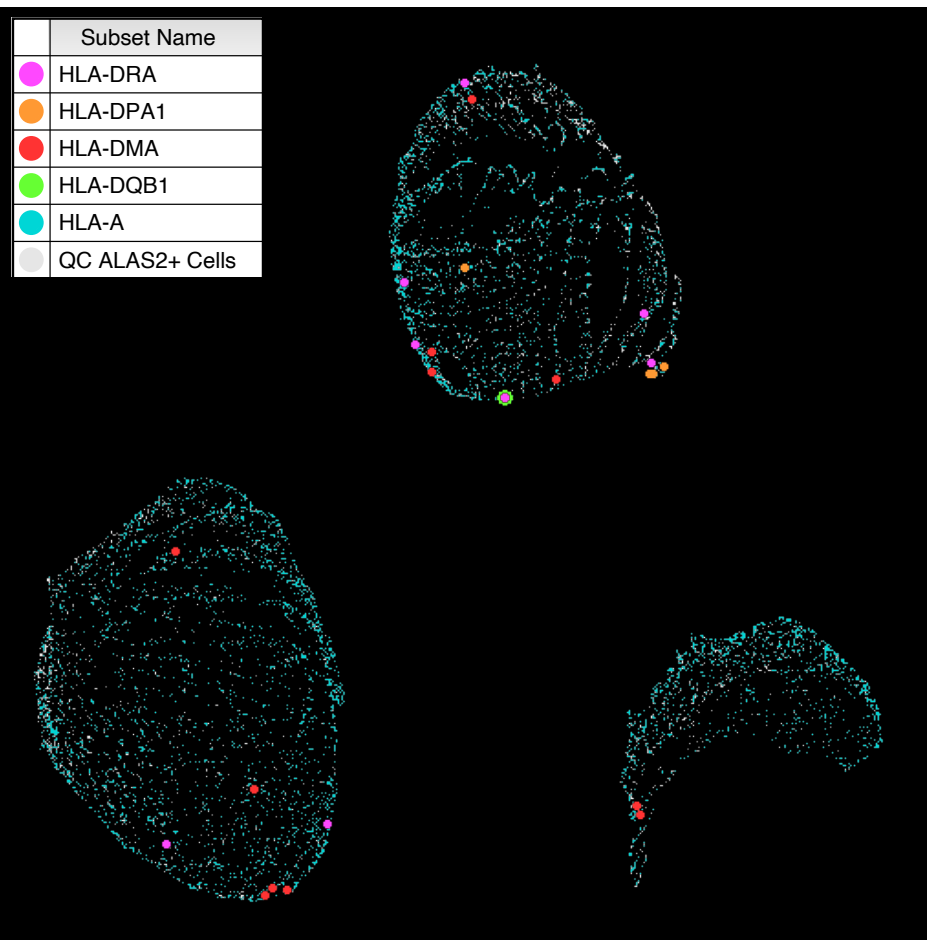

IFNA1

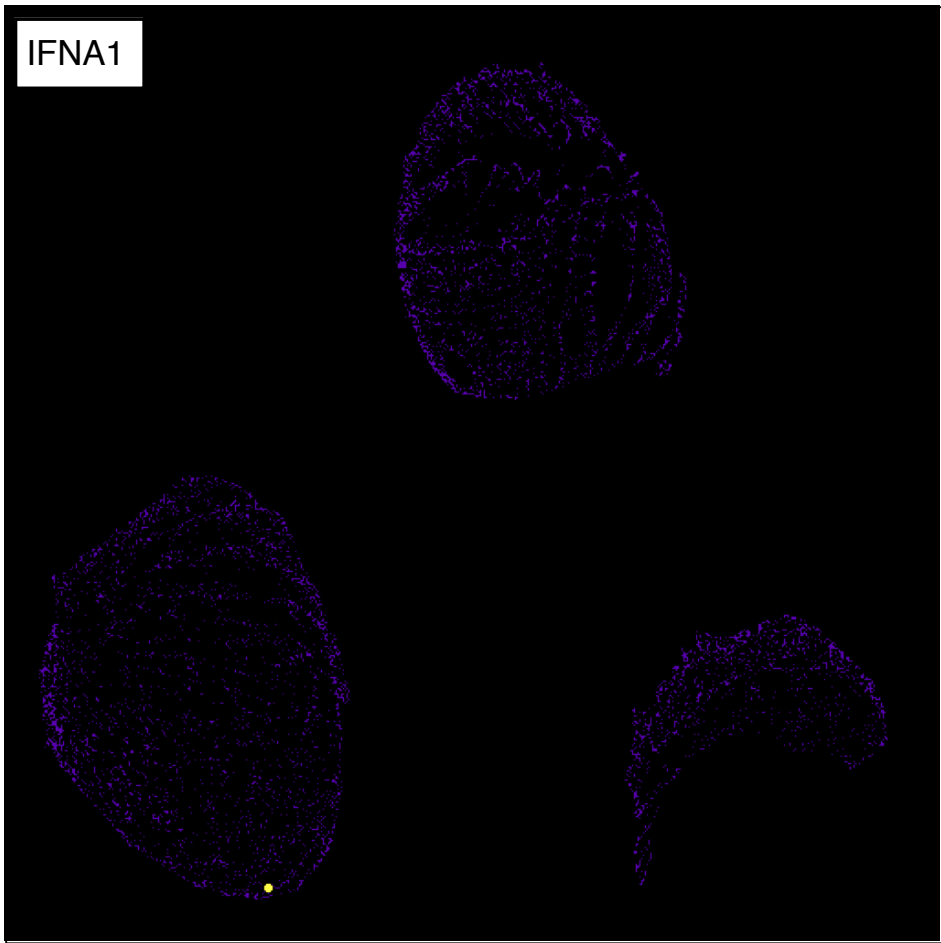

Defensin 4

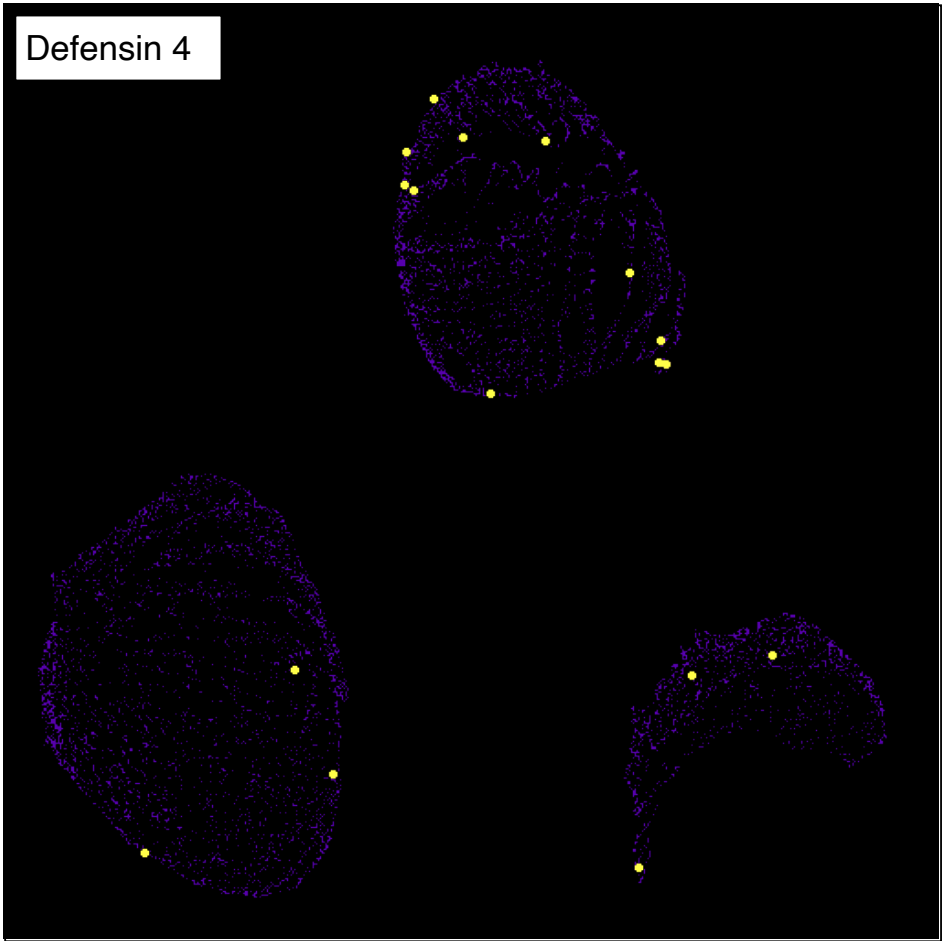

IL15

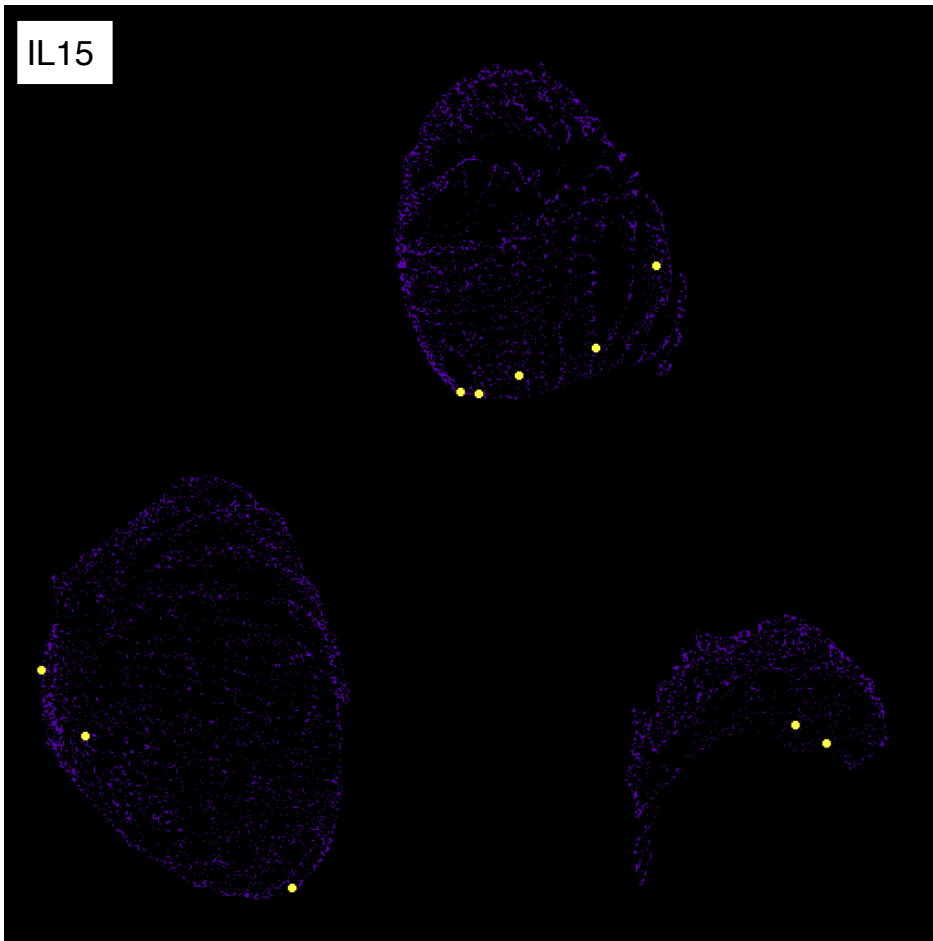

IL18

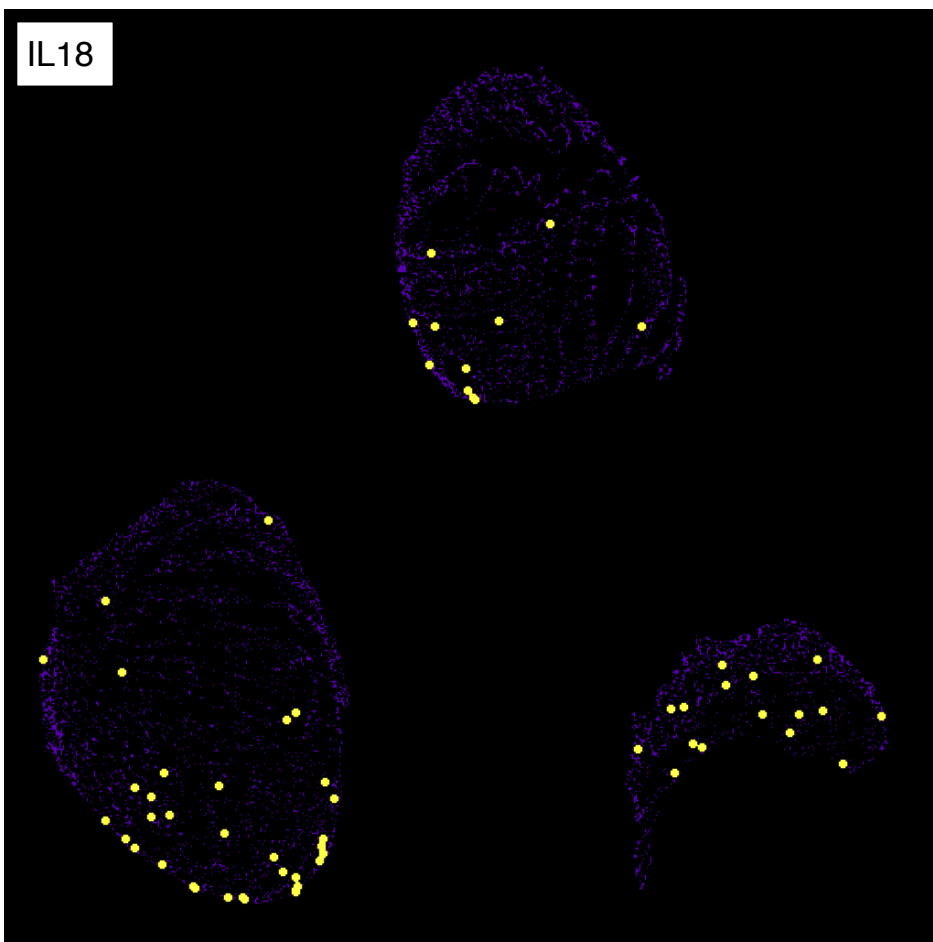

IL1B

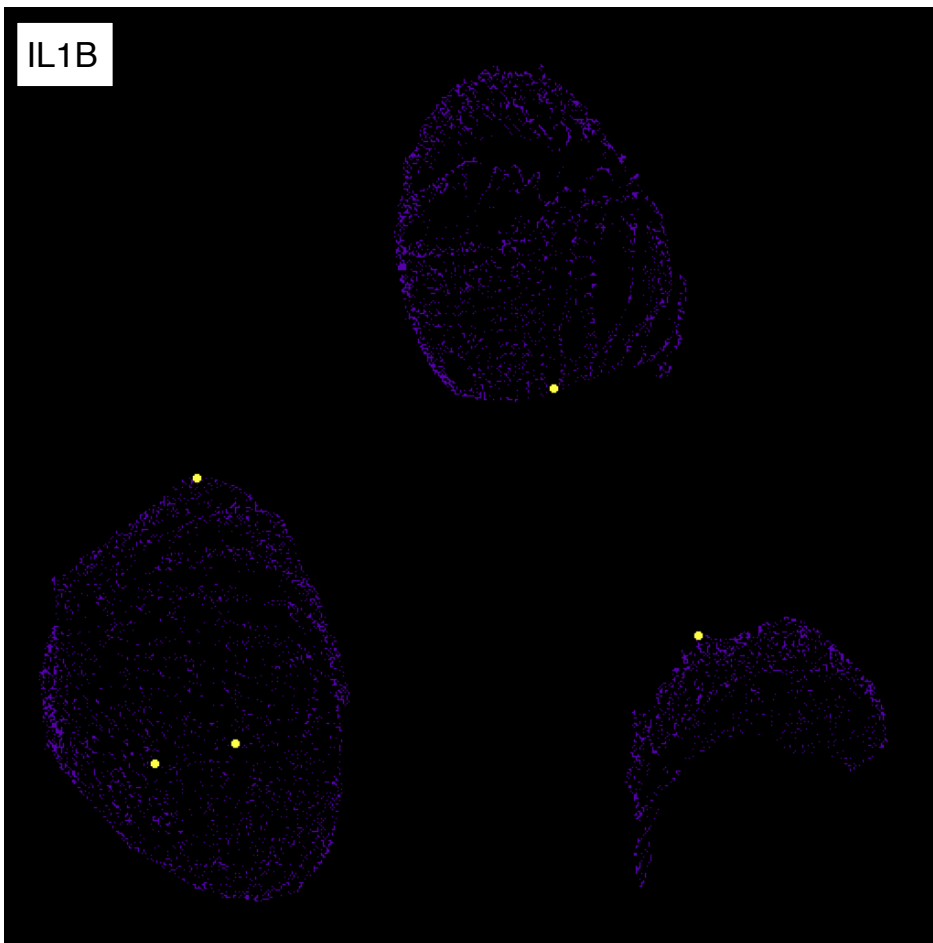

IL33

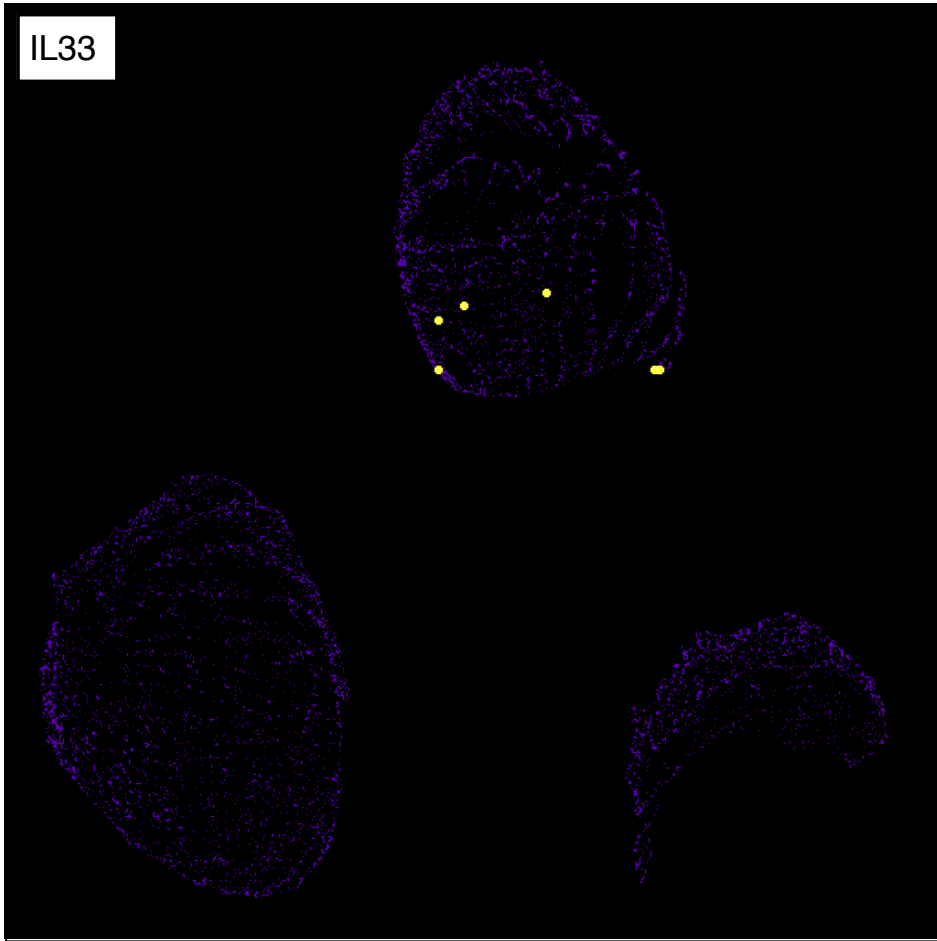

TGFB3

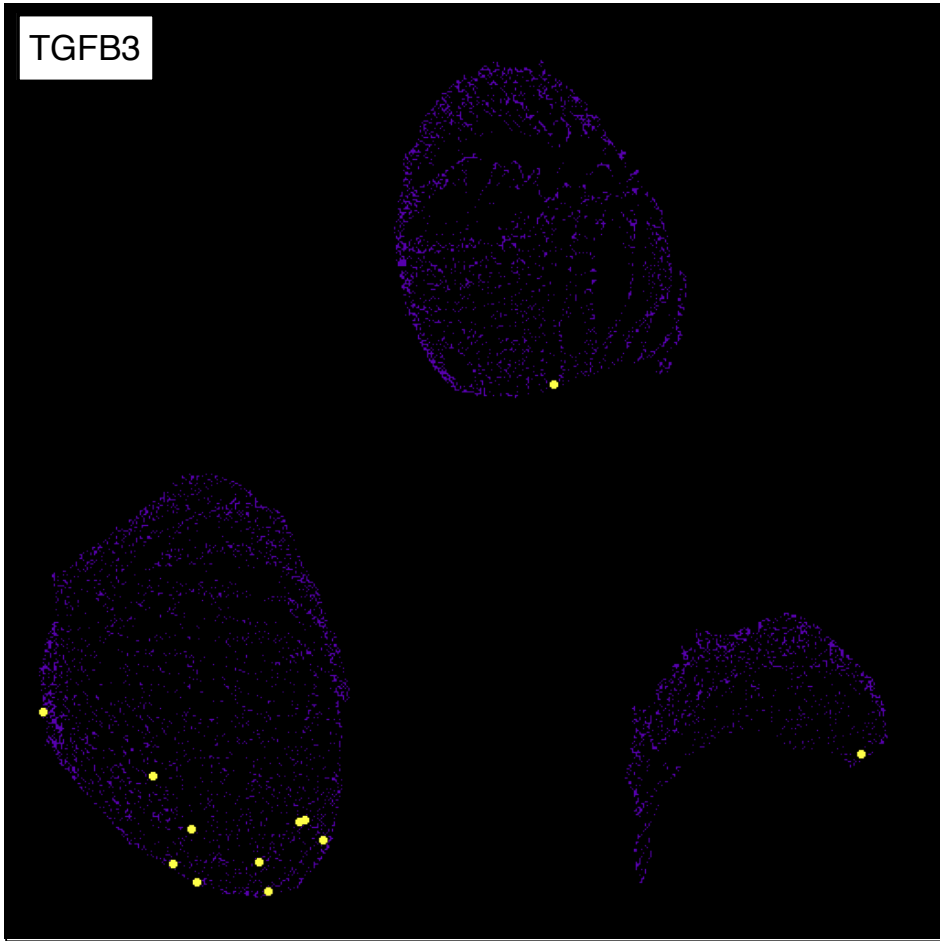

TNF

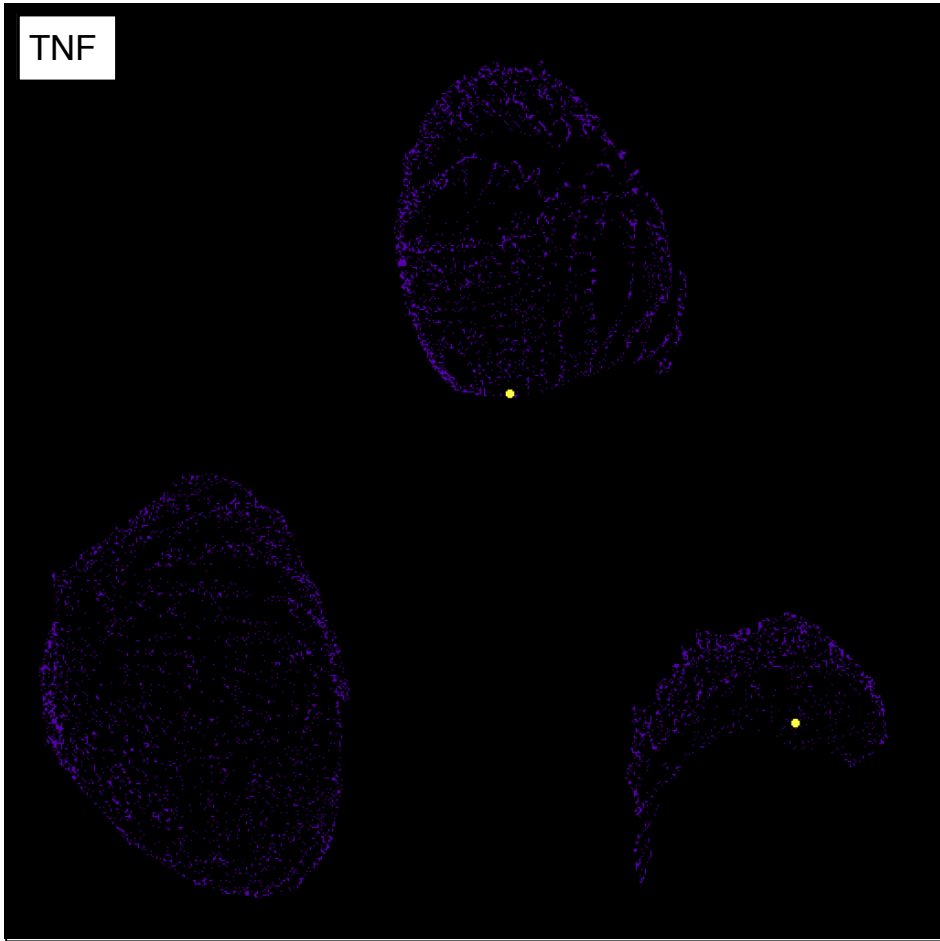

TRAIL

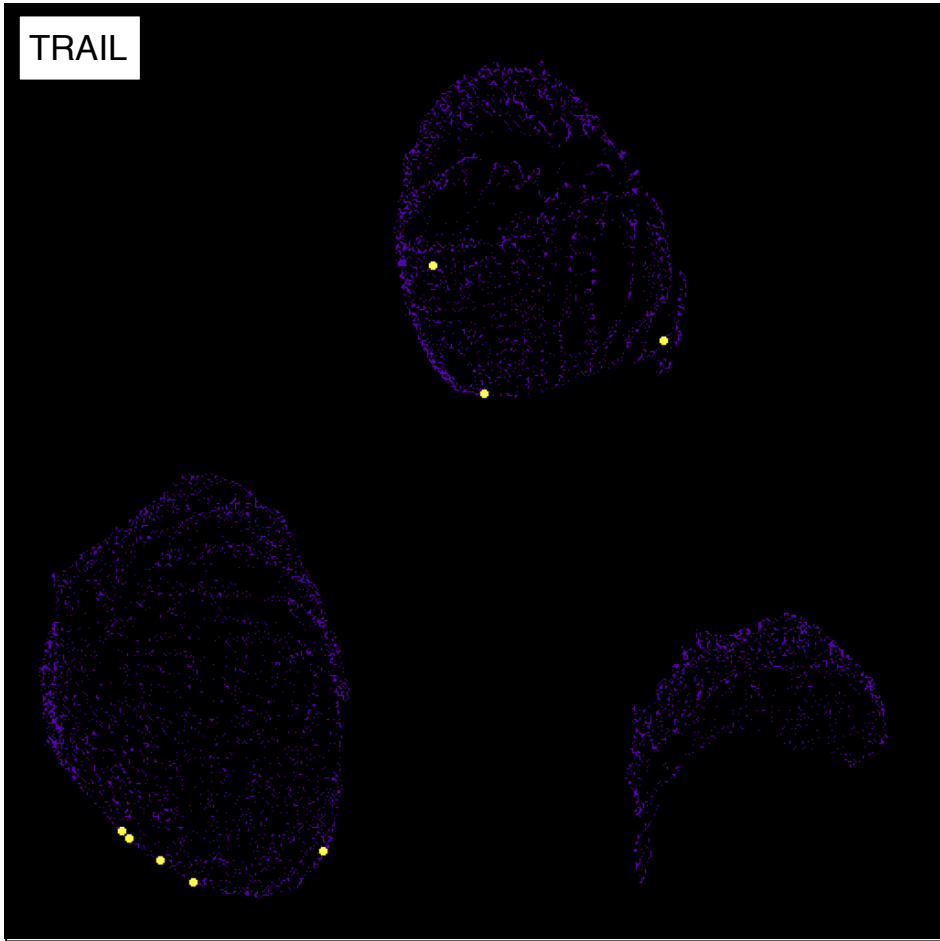

CXCL1

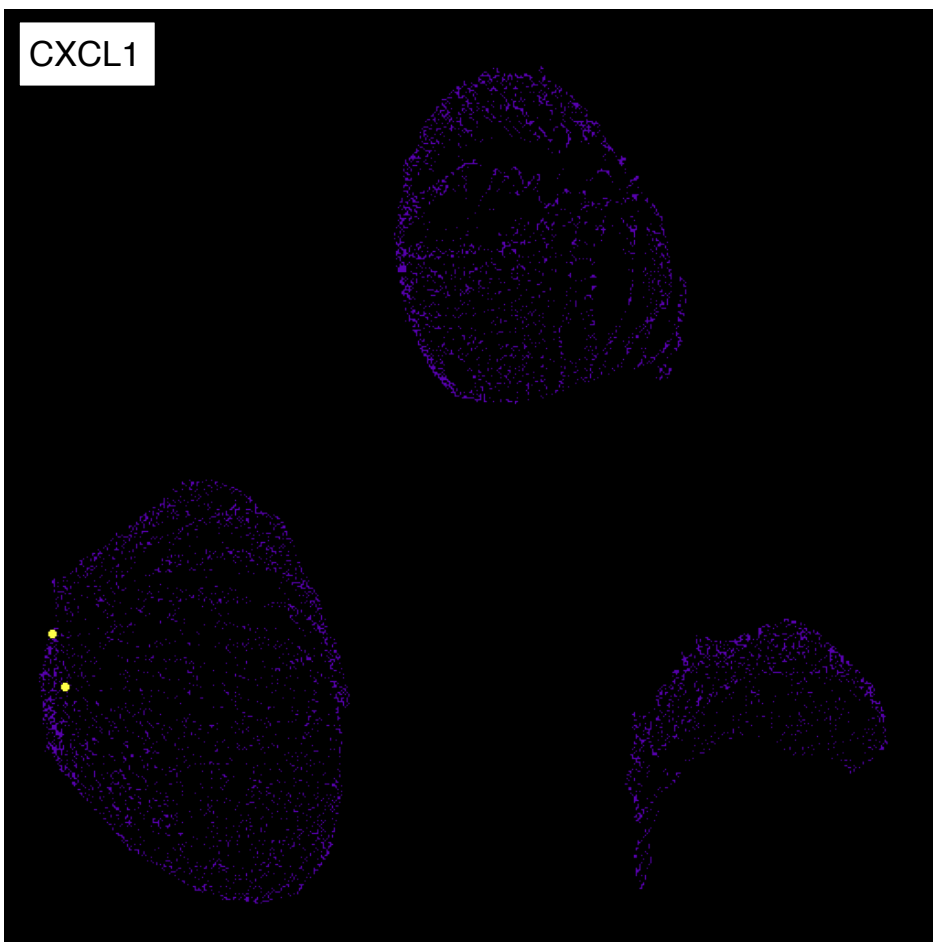

CXCL2

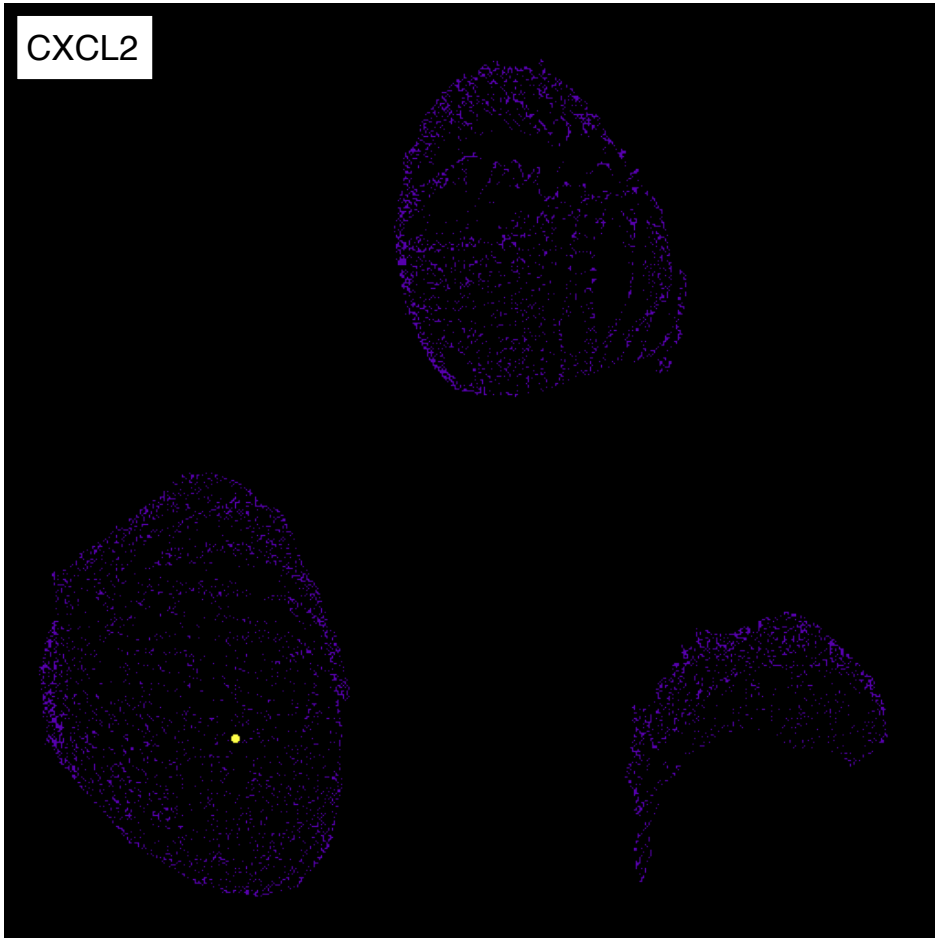

CXCL3

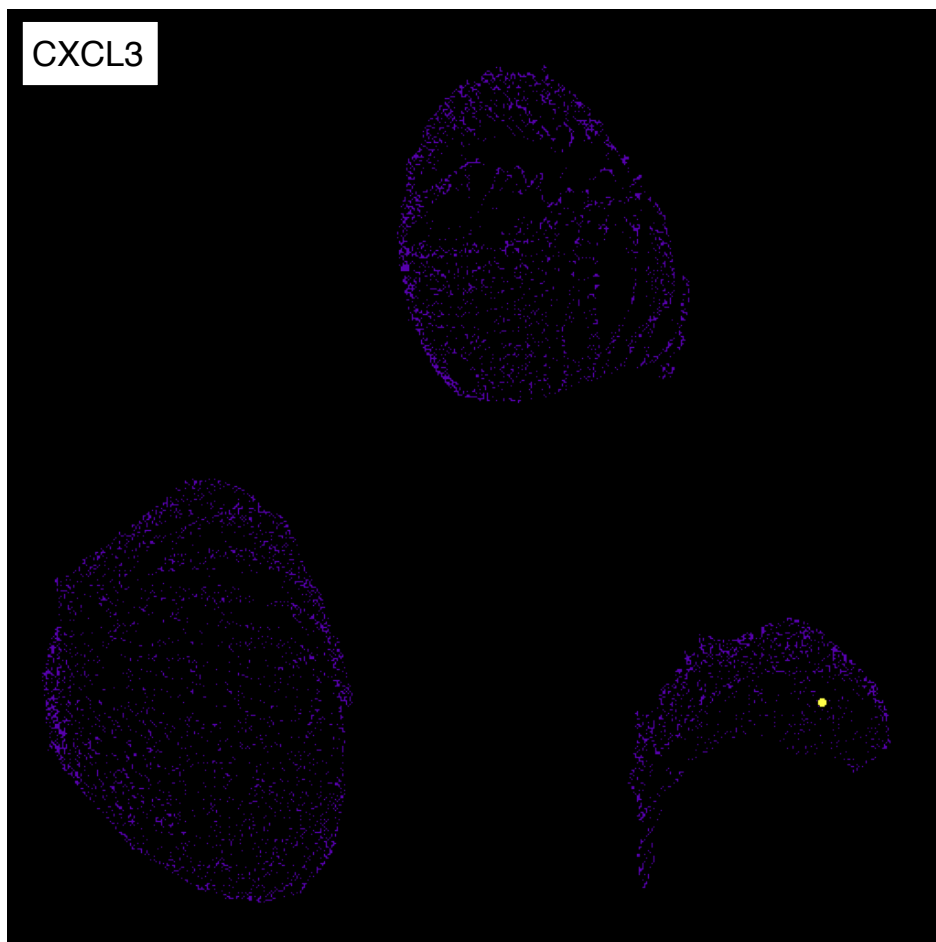

CXCL16

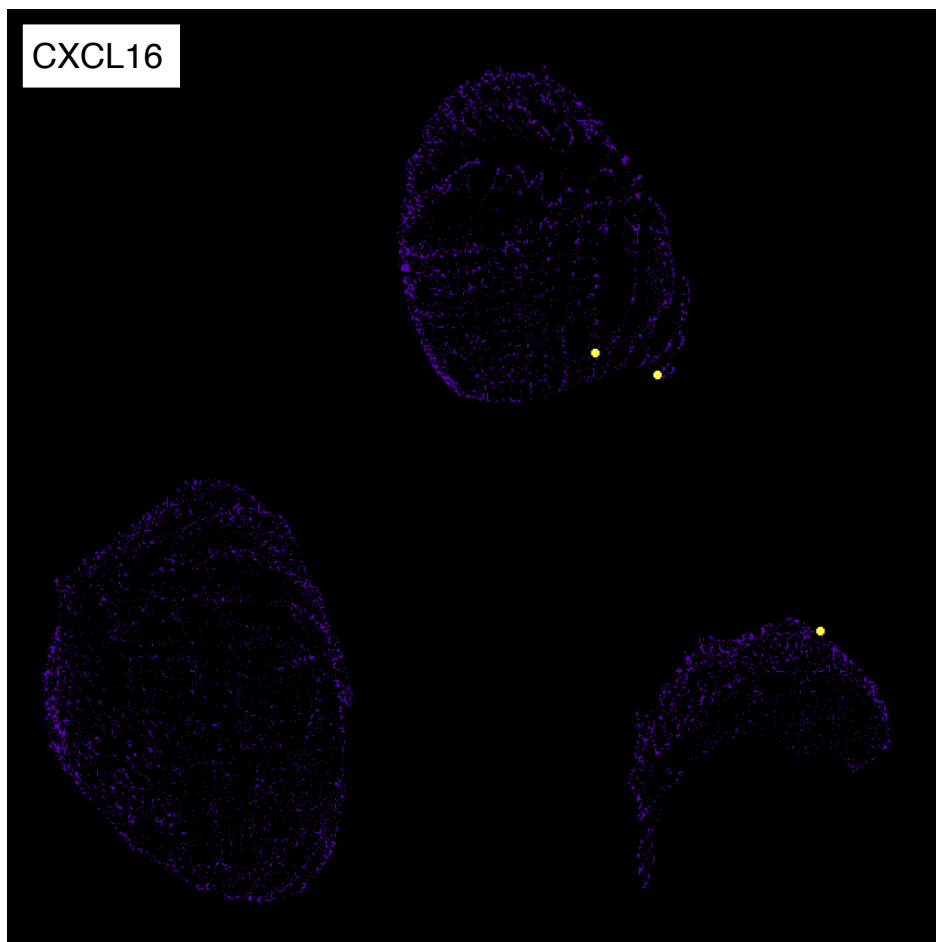

IL12A

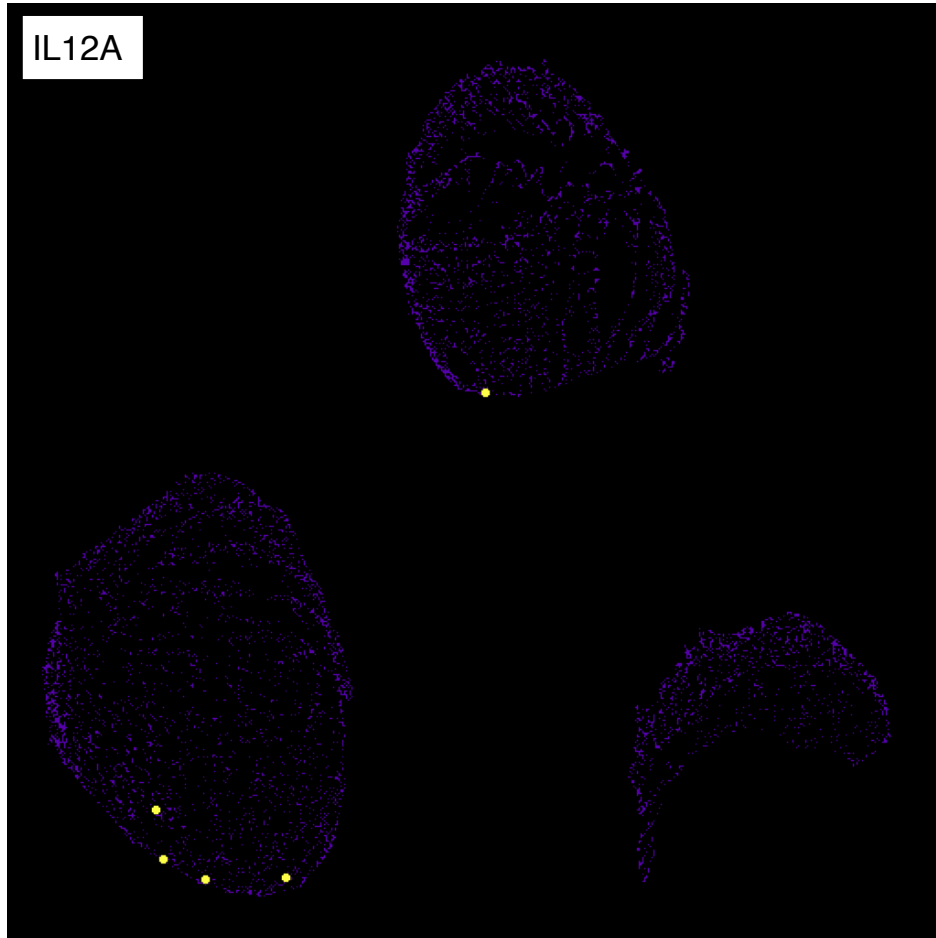

Supplement: Supplementary file 1 [file cells-11-03537-s001.zip › file S1-ABM, CB and FLP NECs' minor cytokine and chemokine gene expression.pdf]
